# The Predictive Properties of Violence Risk Instruments May Increase by Adding Items Assessing Sleep

**DOI:** 10.3389/fpsyt.2019.00323

**Published:** 2019-05-09

**Authors:** Knut Langsrud, Arne Vaaler, Gunnar Morken, Håvard Kallestad, Roger Almvik, Tom Palmstierna, Ismail C. Güzey

**Affiliations:** ^1^Department of Psychiatry, St. Olavs University Hospital, Trondheim, Norway; ^2^Department of Mental Health, Faculty of Medicine and Health Sciences, NTNU–Norwegian University of Science and Technology, Trondheim, Norway; ^3^Forensic Research Unit, Brøset, St. Olavs University Hospital, Trondheim, Norway; ^4^Department of Clinical Neuroscience, Centre for Psychiatric Research, Karolinska Institutet, Stockholm, Sweden

**Keywords:** prediction, sleep, aggression, violence, psychiatry, inpatients

## Abstract

**Background:** The psychometric instruments developed for short-term prediction of violence in psychiatric inpatients do not include variables assessing sleep. Disturbances in sleep may precede aggression in this setting. We investigated whether adding information on sleep improved the predictive properties of the Brøset Violence Checklist (BVC).

**Methods:** The study population consists of all patients admitted to a psychiatric intensive care unit (PICU) over a 6-month period who were hospitalized for at least one night (*n* = 50). Sleep observed by staff (521 nights), behavior assessed with the BVC (433 days), and aggressive incidents recorded by the Staff Observation Scale-Revised (*n* = 14) were included in the analysis.

**Results:** The ability of the BVC to predict aggressive incidents improved from AUC_ROC_ 0.757 to AUC_ROC_ 0.873 when a combined sleep variable including both sleep duration and night-to-night variations of sleep duration was added to the BVC recordings. The combined sleep variable did not significantly predict aggressive incidents (AUC_ROC_ 0.653, *p* = 0.051).

**Conclusions:** A sleep disturbance variable improves the predictive properties of the BVC in PICUs. Further studies of sleep duration, night-to-night variations in duration of sleep, and aggression are needed.

## Introduction

Predicting aggressive behavior is important in acute and emergency psychiatry (1, 2). The Brøset Violence Checklist (BVC) has been developed to assess the risk of imminent aggressive behavior in psychiatric inpatients (3–5). It has a short-term perspective (4–6), and it is used in emergency facilities in a number of countries (6–10).

The predictive properties of the BVC have been documented in different psychiatric facilities including acute and emergency departments (6–10). Although the BVC is a successful predictor of aggressive behavior, its main focus is the patients’ immediate behavior, such as irritability and the display of verbal or physical threats by the patient (3, 4). One variable that has the potential to affect violent behavior directly, which is not included in the BVC, is sleep (11).

Losing sleep may lead to changes in cognitive functions, emotional regulation, and control of behavioral responses (11–13). Although the exact relationship between sleep and aggression is not clear, several studies have indicated an association between them (11). In a study of combat veterans, those who reported sleep duration of less than 6 h had the strongest association between combat exposure and posttraumatic stress, aggression, and risky behavior (14). An inverse association between hours of sleep and aggression has been found in youths (15). In individuals with low-functioning autism, prediction of daytime challenging behavior, including aggression, was strongly driven by sleep variability (16). In an earlier study, we also found an association between disturbance in sleep and aggression the next day in a psychiatric intensive care unit (PICU) (17). We have not found any studies examining whether sleep items or stability of sleep can be used as a short-term predictor of aggression.

The aim of this study was to investigate whether adding items assessing sleep duration and night-to-night variations in sleep duration between two consecutive nights increased the predictive properties of the BVC in a population of patients in a PICU.

## Methods

### Setting

The Norwegian psychiatric health care system is publicly funded and catchment-area based. All patients from the catchment area in need of acute psychiatric inpatient services are admitted to the hospital. At the time of the inclusions, the Department of Psychiatry at St Olav’s University Hospital had 700 admitted patients (≥18 years) each year from a catchment area with 140,000 inhabitants.

The PICUs in the Department of Acute Psychiatry are separated parts of the closed psychiatric acute wards, and each has a sitting room, eating area, bathroom, and patient rooms. There are always allocated nurses in the PICU. The nurses remain with the patients and observe them continuously or at 3- to 30-min intervals, depending on the state of the patients. Details about the catchment area, hospital, PICU, and the assessments have been published previously (17–19).

All admitted patients that stayed for at least one night in the PICU during a 6-month period were included in the study.

The diagnoses were set according to the World Health Organization’s International Classification of Diseases version 10 (ICD-10) criteria for research (20).

### Assessments

#### Sleep Variables

The times the patients were observed to sleep were recorded both in the medical records, and in a separate sleep diary, with one column for each 24-h period and a square for every 30-min block from 12:00 noon to 12:00 noon, by the nurses in PICU. The staff was instructed to record if a patient was considered “to be sleeping” according to their own clinical judgement. The nurses were in the room with the patients or observed them through the door every 3–30 min, depending on the state of the patients.

In calculating the night-to-night variations in sleep duration, the absolute values of the differences in sleep duration between two consecutive nights were used.

To make the sleep items compatible with a checklist and to retain the dimension aspect of sleep, we used a three-point scale rather than the two-point scale used in the BVC. The variables used in analyzing sleep duration, night-to-night variations in sleep duration between two consecutive nights, and the combined variable of both, sleep disturbance, were defined on a 0–2 point scale. Sleep duration for 6 h or more was defined as 0; between 4 and 6 h, 1; and 4 h or less, 2. Night-to-night variations in sleep duration for 2 h or less was defined as 0; between 2 and 4 h, 1; and 4 h or more, 2.

Sleep disturbance was defined as: (sleep duration + night-to-night variation in sleep duration)/2, or only the value from the sleep duration if the patient had only one night of stay.

#### Brøset Violence Checklist

The BVC is an observer-rated instrument predicting imminent (a 24-h perspective) aggressive incidents among psychiatric inpatients (3, 4). The hospital’s staff has experience in the use of this instrument in both daily clinical practice and research (9, 18). After being with the patients for about 1 h on each shift, the nurses rate the six-item checklist of behavior: being confused, irritable, boisterous, verbally threatening, physically threatening, and/or attacking objects. The BVC has a scoring range from 0 to 6. In the present study, the highest of the morning or evening BVC scores was used. When an aggressive incident occurred, only the BVC scores prior to the incident that day were included.

#### New Brøset Violence Checklist Sleep Score

By adding the sleep disturbance score to the BVC sum score the day after sleep, we calculated a new combined BVC-sleep score with a range from 0 to 8.

#### Staff Observation Scale

Aggressive incidents were recorded with the Staff Observation Scale-Revised (SOAS-R) (21, 22). The nurses witnessing an incident filled in the form. The SOAS-R captures different aspects of aggressive incidents: provocation, means, target, consequences for victims, and measures taken to stop the aggression.

#### Statistics

SPSS version 24 was used in the statistical analyses. A *p* value < 0.05 was employed as significant. Normally distributed data were analyzed with Students’ *t* test. The Mann–Whitney *U* test was used to analyze data that were not normally distributed. Receiver operating characteristic curve (ROC curve) and area under the curve (AUC) with confidence intervals (CIs) were used to analyze the variables’ ability to predict aggressive incidents. No missing data replacement was used (23).

## Results

### Characteristics of the Sample

A total of 40 patients with 50 admissions and 521 nights were included in the study. Females had 21 admissions and males had 29. The mean age in the sample was 41.7 (SD ± 17.4) years. Patients with a main diagnosis of schizophrenia (F20–29) made up the majority of the admissions (*n* = 21) and nights (*n* = 381). Patients with mood disorders (F30–39) had 13 admissions and 99 nights, patients with substance abuse (F10–19) had 9 admissions and 19 nights, patients with organic disorders (F00–09) had 6 admissions and 20 nights, and 1 patient with other diagnoses (F40–99) had 2 nights.

The mean sleep duration was 9.0 (SD ± 2.9) h and the mean absolute difference in sleep duration from night-to-night was 2.0 (SD ± 1.9) h. The median number of nights per admission in the PICU was 3 (IQR 1.75–11.0). Of the 433 BVC forms, 355 had BVC = 0, categorized as small risk; 69 had BVC = 1–2, categorized as moderate risk; and 19 had BVC > 2, categorized as high risk of violence. Fourteen aggressive incidents (SOAS-R) from six patients were recorded. Sleep data were missing on 12 nights and BVC data were missing on 78 days.

### Receiver Operating Characteristic Curve

**Figure 1:**
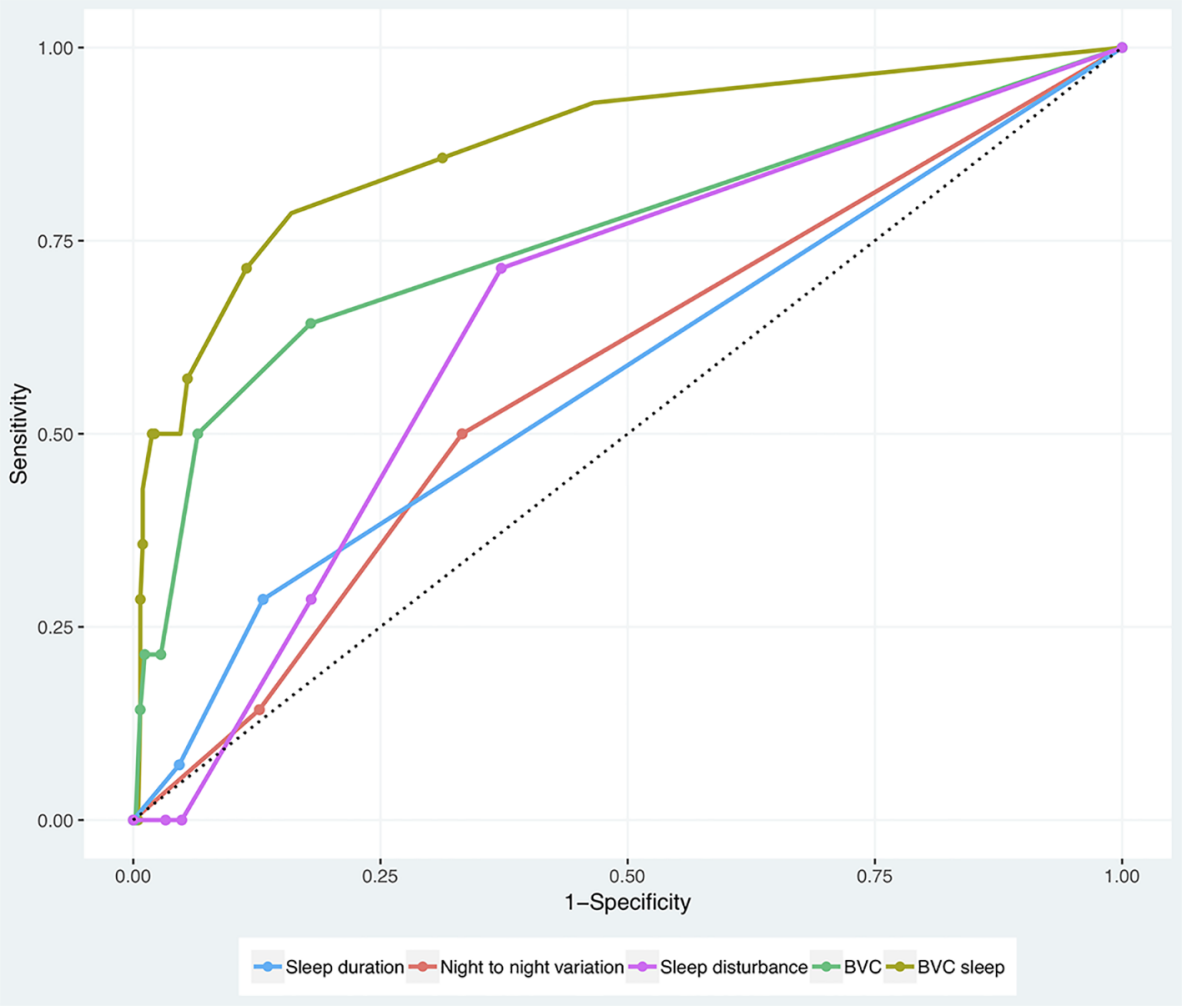
Receiver operating characteristic (ROC) analyses of sleep duration, night-to-night variation in sleep duration, sleep disturbance (combined variable of sleep duration and night-to-night variations), Brøset Violence Checklist (BVC), and BVC-sleep (a combination of BVC and sleep disturbance).

The ROC analyses can be seen in **Figure 1** and the AUC_ROC_ can be seen in **Table 1**. BVC gave an AUC_ROC_ value of 0.757. The new combined BVC-sleep score gave an AUC_ROC_ value of 0.873, improving the BVC score. Both BVC and BVC-sleep AUC_ROC_ values were highly significant. None of the sleep variables alone gave a significant AUC_ROC_ value.

**Table 1:** Area under the curve (AUC_ROC_) for sleep duration, night-to-night variation in sleep duration, sleep disturbance (a combined variable of both sleep duration and night-to-night variations), Brøset Violence Checklist (BVC), and BVC-sleep (a combination of BVC and sleep disturbance).

	Missing	AUC	95% CI	Sign^a^
Sleep duration	12	0.575	(0.412–0.738)	0.337
Sleep night-to-night variations	68	0.576	(0.424–0.728)	0.335
Sleep disturbance	18	0.653	(0.520–0.786)	0.051
BVC	78	0.757	(0.601–0.914)	0.001
BVC-sleep	84	0.873	(0.767–0.981)	0.000

CI, confidence interval.

a Null hypothesis: true area = 0.05.

## Discussion

In this prospective study, the BVC was shown to be sensitive in predicting aggressive incidents, and adding sleep disturbance to the BVC scores improved its predictive capacity further. Neither short duration of sleep nor high night-to-night variations in duration of sleep predicted aggressive incidents at the same level as BVC scores alone. As far as we are aware, only a previous study from our group has explored the relationship between sleep and aggression in acute or emergency psychiatry services (17). That study provided support for a possible association between sleep disturbances and aggression. The current study indicates that observations of sleep improve the sensitivity of the BVC.

The BVC is a recommended instrument for the short-term prediction of aggressive incidents. It can also be used as a decision-making tool for seclusion in the PICU (6). In this study, the ability of the BVC to predict aggressive incidents (AUC_ROC_ 0.757, *p* = 0.001) was somewhat lower than or comparable to earlier studies (4, 8, 9), but the prediction of aggressive incidents was improved by adding the variable for sleep (AUC_ROC_ 0.873, *p* = 0.000). A study that added Visual Analogue Scale ratings of subjective perception of risk for a physical attack to the BVC (8) improved the BVC at a comparable level.

Adding sleep information to the BVC requires extra effort, especially if the sleep information is not routinely recorded during treatment in the PICU. In the study PICUs, collection of sleep information was a part of the standard treatment protocol and therefore added little to the workload of the staff. Violent behavior is common in acute psychiatric wards. It is found that one out of five patients commit an act of violence, 75–100% of the nurses have been assaulted by patients, and annually one-third of the total nursing costs are connected to managing violence and aggression (1, 2). These high numbers make even a marginal improvement in predicting aggression valuable for patients and staff, in order to improve safety (8, 10).

In the present study, neither sleep duration (AUC_ROC_ 0.575, *p* = 0.337) nor night-to-night variations in sleep duration between two consecutive nights (AUC_ROC_ 0.576, *p* = 0.335) predicted aggressive incidents the next day. When the sleep duration and variability of sleep duration between two consecutive nights were combined in the new sleep disturbance variable, a numerically larger area under the curve (AUC_ROC_ 0.653, *p* = 0.051) was found, but the difference did not reach statistical significance. The reason for this might be the complex nature of aggressive behavior (24), and that sleep duration and night-to-night variability in sleep duration capture different aspects of sleep disturbance when analyzed as isolated predictors. By adding night-to-night variation (25), we may assess disturbances in sleep better than when analyzing sleep duration alone.

Earlier studies suggest that sleep deprivation leads to a complex set of changes such as increased sensitivity to identification of negative stimuli, increased hostile attributions, and loss of inhibition of context-inappropriate responses. Reduced top-down control of emotional signals, poor affect regulation, in addition to decreased emotional intelligence may contribute to the overall aggressive behavior (11–13). These neurobiological changes are complex and may not be captured completely by the BVC’s six-item checklist of behavior.

### Strengths and Limitations

The naturalistic setting in the PICU, inclusion of all admitted patients from the catchment area, and the use of standardized measures for risk behavior and aggressive incidents are the major strengths of the present study.

The currently used “observed sleep” by the nurses has both strengths and weaknesses (26). It is the standard measure for sleep in most psychiatric departments in daily, clinical practice. It is therefore already implemented. However, the nurse-based observations of sleep may be prone to errors, such as misjudging what has been observed, not noticing when patients may be awake, or being interrupted in the observations, giving reduced report quality. The clinical observation by nurses is not as objective as polysomnography or actigraphy, assessments tools that are not possible to use in the PICUs because of security, cost, and ethical reasons. However, some studies document satisfactory correlations between these different measures (26–28). Another weakness in the study is the lack of registered information on other risk factors for aggression such as involuntary admission, alcohol abuse, and history of violence.

## Conclusions

After adding sleep disturbance to the BVC, an instrument for short-term prediction of violent behavior from inpatients, both the specificity and the sensitivity of the instrument improved. The combined variable of sleep duration and night-to-night variations in duration of sleep (sleep disturbance) seems to have a promising capacity to contribute to prediction. Further studies of sleep duration, night-to-night variations in duration of sleep, and aggression are necessary.

## Data Availability Statement

Data are available upon request to the corresponding author.

## Ethics Statement

All the data used in this study were collected as part of ordinary routine, clinical practice in the department. Patients acutely admitted to PICUs have limited capacity to consent in taking part in clinical studies. This is due to the mixture of psychiatric and behavioral challenges. The patients in this study were thus included without giving an informed, written consent. The study including the inclusion procedures was approved by the Regional Committee for Medical and Health Research Ethics, Central Norway.

## Author Contributions

KL contributed to the planning, data management and analyses, interpretation of the results, and writing. AV, GM, and RA contributed to the planning, interpretation of the results, and writing. HK and TP contributed to the interpretation of the results and writing. ICG contributed to the analyses, interpretation of the results, and writing. All authors read and approved the final manuscript.

## Funding

Funding was obtained from St. Olavs University Hospital, Trondheim, Norway.

## Conflict of Interests Statement

The authors declare that the research was conducted in the absence of any commercial or financial relationships that could be construed as a potential conflict of interest.

## Abbreviations

AUC, area under the curve; BVC, Brøset Violence Checklist; CI, confidence interval; PICU, psychiatric intensive care unit; SOAS-R, Staff Observation Scale-Revised; ICD-10, Diagnoses according to World Health Organization’s International Classification of Diseases, version 10; ROC curve, receiver operating characteristic curve; SD, standard deviation.
